# BMI is independently associated with ARDS, sepsis and multiorgan failure after major trauma—results of a high-volume retrospective observational cohort study

**DOI:** 10.1186/s13049-026-01603-7

**Published:** 2026-03-27

**Authors:** B. Erdle, J. Mangold, J. Kalbhenn, T. D. Diallo, F. C. Wagner, J. Maier, H. Schmal, N. Mühlenfeld

**Affiliations:** 1 Department of Orthopedics and Trauma Surgery, Medical Centre – Albert‑Ludwig’s University Freiburg, Faculty of Medicine, Albert‑Ludwig’s University of Freiburg, Hugstetter Straße 55, Freiburg, D‑79106 Germany; 2 Department of Anesthesiology and Critical Care, Medical Centre – Albert‑Ludwig’s University Freiburg, Faculty of Medicine, Albert‑Ludwig’s University of Freiburg, Freiburg, Germany; 3 Department of Diagnostic and Interventional Radiology, Medical Centre – Albert‑Ludwig’s University Freiburg, Faculty of Medicine, Albert‑Ludwig’s University of Freiburg, Freiburg, Germany; 4 Department of Orthopedic Surgery, University Hospital Odense, Odense, Denmark

**Keywords:** Trauma, Obesity, BMI, Sex, ARDS, Multiorgan failure, Sepsis, Pneumonia, Mortality

## Abstract

**Background:**

Obesity is increasingly prevalent among trauma patients and has been proposed to influence post-injury inflammatory responses and organ dysfunction. However, evidence on the independent effect of body mass index (BMI) on in-hospital complications after major trauma remains conflicting. This study aimed to determine whether BMI is an independent predictor of specific adverse clinical outcomes in a large cohort of adult trauma patients.

**Methods:**

This retrospective cohort study analyzed associations between BMI and adverse clinical outcomes (ARDS, pneumonia, sepsis, multiorgan failure (MOF) and mortality) among adult trauma patients at a German level-I trauma center between 2018 and 2024. Inclusion criteria were trauma leading to an Injury Severity Score (ISS) ≥ 9 and/or admission to the intensive care unit. Multivariable logistic regression adjusted for BMI, age, sex, ASA and ISS.

**Results:**

A total of 1514 patients were included. Adjusted multivariate regression revealed BMI as being independently associated with an increased risk of ARDS (aOR 1.09 per kg/m^2^, 95% CI 1.01–1.16; *p* = 0.025), sepsis (aOR 1.05, 95% CI 1.00–1.10; *p* = 0.048) and MOF (aOR 1.10, 95% CI 1.04–1.17; *p* < 0.001), but not with pneumonia or mortality. Categorical BMI analysis identified obesity (> 30 kg/m^2^) as a clinically relevant non-linear cut-off point.

**Conclusions:**

A higher BMI was independently associated with ARDS, sepsis and MOF, but no independent association with mortality was detected in this cohort of adult trauma patients. This suggests that BMI may function as a clinically relevant risk marker.

**Supplementary Information:**

The online version contains supplementary material available at 10.1186/s13049-026-01603-7.

## Introduction

Obesity has become a global health challenge, with prevalence rates rising steadily across all age groups and healthcare systems [1]. As obesity rates rise, trauma centers are confronted with a growing number of severely injured patients with elevated body mass index (BMI). The interaction between obesity and traumatic injury is complex and not yet fully understood. Obesity is known to modulate immune responses, impair respiratory mechanics, and alter cardiometabolic function, potentially influencing the trajectory of post-traumatic recovery [2–4].

Previous research has yielded conflicting results regarding the impact of obesity on clinical outcomes. Several studies from recent decades have described increased rates of complications such as acute respiratory distress syndrome (ARDS), infections, and multiorgan failure (MOF) among obese trauma patients [5–10]. Meta analyses have consistently revealed longer intensive care therapy and total in-hospital length of stay [11, 12]. However, other investigations reported similar or even lower mortality in obese versus non-obese injured patients, often referred to as the “obesity survival paradox” [13–16]. More recent analyses in trauma and critical care populations have further highlighted that the association between obesity and post-injury complications is complex and may differ across outcomes: while contemporary trauma studies reported higher complication rates but unchanged mortality in obese patients [17, 18], large-scale critical care meta-analyses reported neutral or even protective associations of moderate obesity with general ICU mortality [19, 20].

Although numerous studies have examined obesity-related risks in injured or critically ill patients, most available evidence does not directly reflect a polytrauma population. Many investigations focus on general ICU cohorts [2, 4, 19–23] or on trauma patients with specific injury patterns, most commonly isolated thoracic trauma [24, 25] or abdominal trauma [26], limiting generalizability to complex multisystem injuries. Among polytrauma studies, the majority report BMI-associated differences as ICU or hospital length of stay, or overall mortality [15, 27]. High-quality analyses addressing organ dysfunction or specific complications such as ARDS, MOF or sepsis remain scarce, often constrained by small cohort sizes, single-injury subgroups, or heterogeneous definitions of organ failure [4, 5, 17, 28].

Consequently, the comparability of prior studies to the present investigation is limited, particularly regarding specific early post-traumatic complications.

Very few contemporary trauma studies have explicitly examined obesity in relation to early organ-dysfunction outcomes such as ARDS, MOF [8, 28] or infectious complications, including sepsis or pneumonia [29–31]. Fndings remain heterogeneous even among these, as they report inconsistent associations across BMI categories, injury patterns, and analytic approaches. Most available trauma studies focus primarily on mortality, rather than on clinically significant but non-fatal endpoints such as ARDS, pneumonia, sepsis or multi-organ dysfunction [11, 12]. As a result, robust contemporary data explicitly addressing the relationship between BMI and post-traumatic organ dysfunction remain limited.

These inconsistencies underscore the need for modern trauma-specific analyses evaluating clinically relevant endpoints beyond mortality. The present study therefore aimed to evaluate the BMI’s independent association with major adverse clinical outcomes including ARDS, pneumonia, sepsis and MOF in a large cohort of adult polytrauma patients treated at a Level-I trauma center. We hypothesized that higher BMI would be independently predictive for these risks after trauma, even after adjustment for demographic and injury-related confounders.

## Methods

For this retrospective observational cohort study (ethics approval No. 24–1512-S1-retro), all whole-body trauma CT scans performed at a German level I trauma center between January 2018 and December 2024 were screened to identify eligible adult trauma patients. Inclusion criteria were an Injury Severity Score (ISS) ≥ 9 and/or admission to the intensive care unit (ICU). Non-trauma cases (e.g., medical emergencies undergoing trauma CT for diagnostic reasons), underage patients, and cases with missing or insufficient BMI data were excluded.

The study followed STROBE and RECORD guidelines for observational research.

Data on age, sex, Body Mass Index (BMI), physical status classification according to American Society of Anesthesiologists (ASA), injury severity based on body regional (Abbreviated Injury Scale, AIS) and global Injury Severity Score (ISS) as well as clinical adverse outcomes acute respiratory distress syndrome (ARDS), pneumonia, sepsis, multiorgan failure (MOF) and in-hospital mortality were obtained from electronic health records.

All outcomes were defined according to the institutional and international standards in effect during the study period, applying the Berlin definition for ARDS [32], Sepsis-3 criteria for Sepsis [33] and the Sequential Organ Failure Assessment (SOFA) score > 2 in at least two organ systems for MOF. Pneumonia was diagnosed by clinical signs and radiological evidence. Death from any cause during index hospitalization accounted for mortality. Body mass index (BMI), calculated as weight/height^2^ (kg/m^2^), was analyzed both continuously (kg/m^2^) and categorically (non-obese < 30 kg/m^2^ vs. obese ≥ 30 kg/m^2^ and according to WHO strata).

### Statistical analysis

All data were tested for normality distribution using the Kolmogorov–Smirnov test. Continuous data are reported as mean ± standard deviation (SD) for improved interpretability, even where distributions were non-normal. Group comparisons however were made using the appropriate Mann–Whitney U test for non-parametric data to ensure statistical robustness. Categorical variables were compared using Chi-square tests. Multivariable logistic regression models were constructed for each outcome using the a priori defined covariates BMI, ISS, ASA, age and sex. Complete-case analysis was applied for each model. Results are presented as crude or adjusted odds ratios (OR/aOR) with 95% confidence intervals (CI). Model discrimination was quantified using the area under the receiver operating characteristic curve (AUC). Model calibration was assessed with the Hosmer–Lemeshow goodness-of-fit test (HL). Model fit and parsimony were summarized using the Akaike Information Criterion (AIC). Small-sample robustness was ensured using Firth’s penalized logistic regression to mitigate overfitting concerns. As an additional sensitivity analysis, we modeled ARDS including BMI, age, sex, ASA, and thoracic injury severity with and without ISS, to assess robustness.

Multicollinearity was ruled out using Variance Inflation Factor (VIF). Statistical significance was defined as *p* < 0.05. All statistical analyses were performed using IBM SPSS Statistics (version 30.0; IBM Corp., Armonk, NY, USA) and R (version 4.5.2; R Foundation for Statistical Computing, Vienna, Austria).

## Results

### Baseline characteristics

**Fig. 1:**
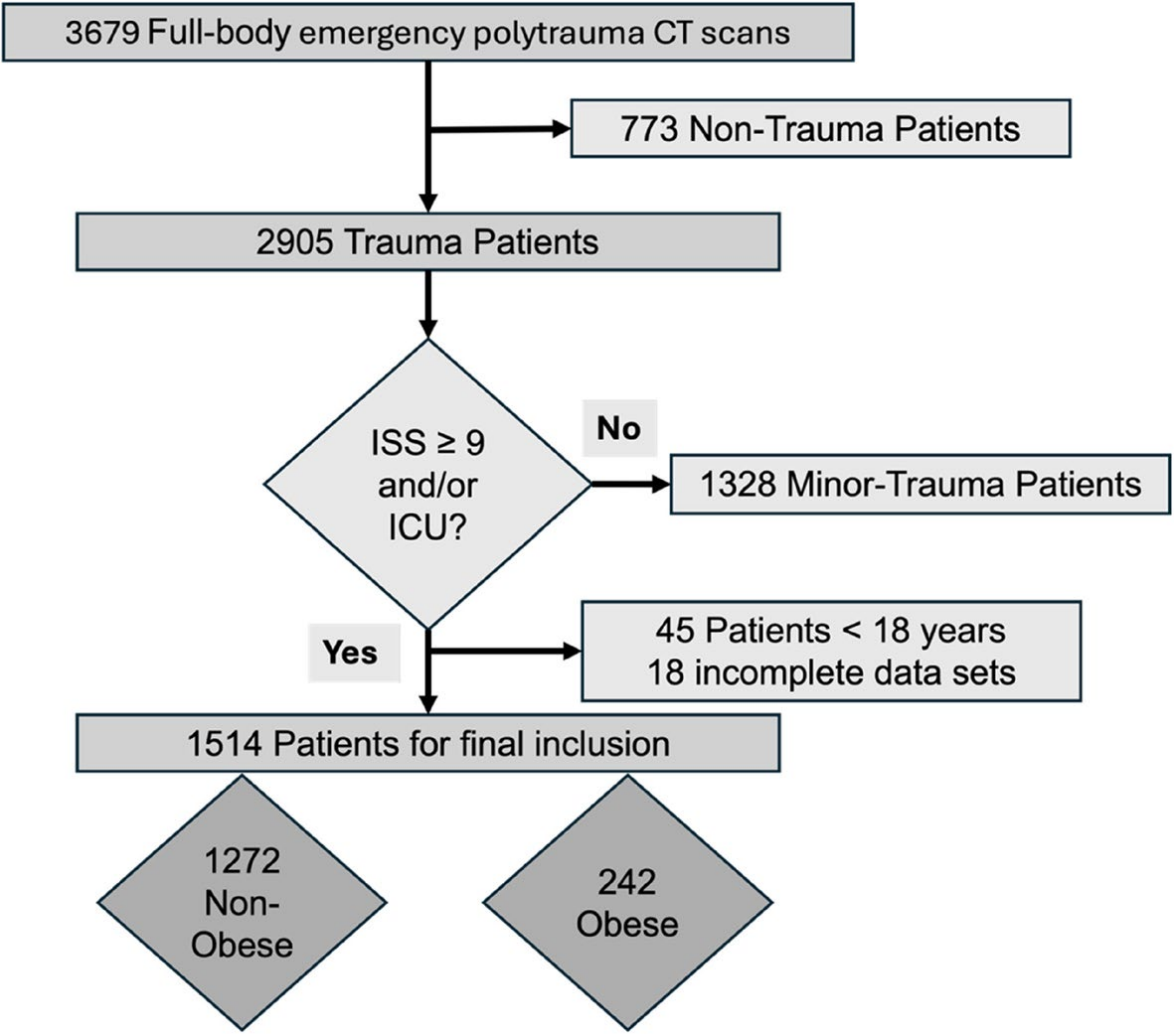
Flow diagram of patient inclusion

A total of 1577 patients met our inclusion criteria. Of these, 45 underage patients and 18 patients with missing or incomplete BMI data were excluded, resulting in a final study cohort of 1514 patients (Fig. 1). We had access to complete data on patient and injury characteristics as well as all predefined adverse outcomes for this cohort.

Stratified to WHO categories, 2.1% (32) of patients were underweight (BMI < 18.5 kg/m^2^), 44.8% (679) normal weight (BMI 18.5–24.9 kg/m^2^) and 37.1% (561) overweight, summarizing 84.0% (1272) as non-obese (BMI < 30 kg/m^2^). Obesity (BMI ≥ 30 kg/m^2^) was present in 16.0% (242) of the cohort, including 10.9% (165) with class I, 4.0% (61) with class II, and 1.1% (16) with class III obesity.

Baseline demographics and injury characteristics are presented in Table 1.

**Table 1:** Baseline Characteristics

Patient and injury characteristics	Overall (*n* = 1514)	Non-obese (BMI < 30 kg/m^2^; *n* = 1272)	Obese (BMI ≥ 30 kg/m^2^; *n* = 242)	*p*-value
BMI, mean ± SD (kg/m^2^)	26.3 ± 4.6	24.8 ± 2.8	34.2 ± 4.1	< 0.001*
Age, mean ± SD (years)	53.1 ± 20.3	52.4 ± 20.7	56.3 ± 17.5	0.003*
Female sex, n (%)	375 (24.8%)	314 (24.7%)	61 (25.2%)	0.815
ASA ≥ 3, n (%)	523 (34.5%)	409 (32.2%)	114 (47.1%)	< 0.001*
ISS, mean ± SD	20.0 ± 11.6	20.2 ± 11.7	18.6 ± 11.0	0.047*
AIS ≥ 3 Head, n (%)	578 (38.2%)	507 (39.9%)	71 (29.3%)	0.003*
AIS ≥ 3 Thorax, n (%)	705 (46.6%)	575 (45.2%)	130 (53.7%)	0.018*
AIS ≥ 3 Abdomen, n (%)	182 (12.0%)	155 (12.2%)	27 (11.2%)	0.732
AIS ≥ 3 Extremity, n (%)	389 (25.7%)	329 (25.9%)	60 (24.8%)	0.788

^*^Statistically significant

Obese patients were significantly older than non-obese patients, exhibited a more prevalent higher ASA and lower mean ISS. They sustained proportionally more severe thoracic injuries, but fewer severe head injuries. The proportion of male and female patients was similar between groups.

Older adults were well represented in the cohort, with 30.4% aged ≥ 65 years and 18.0% aged ≥ 75 years.

### Adverse clinical outcomes

The distribution of clinical complications is illustrated in Table 2. Obese patients had significantly higher unadjusted rates of several key complications such as ARDS, sepsis and MOF. Pneumonia rates and mortality were similar between groups.

**Table 2:** Adverse clinical outcomes

Outcome	Overall (*n* = 1514)	Non-obese (BMI < 30 kg/m^2^; *n* = 1272)	Obese (BMI ≥ 30 kg/m^2^; *n* = 242)	*p*-value
ARDS, n (%)	32 (2.1%)	22 (1.7%)	10 (4.1%)	0.033*
Pneumonia, n (%)	243 (16.0%)	202 (15.9%)	41 (16.9%)	0.687
Sepsis, n (%)	85 (5.6%)	58 (4.6%)	27 (11.2%)	< 0.001*
Multiorgan failure, n (%)	51 (3.4%)	33 (2.6%)	18 (7.4%)	< 0.001*
Mortality, n (%)	160 (10.6%)	133 (10.5%)	27 (11.2%)	0.720

^*^Statistically significant

### Univariable analysis

We conducted univariable logistic regression for every adverse clinical outcome (Supplementary Table 1). Crude BMI as a continuous predictor (per kg/m^2^) demonstrated significant associations with ARDS (OR 1.08, 95% CI 1.02–1.15; *p* = 0.012), sepsis (OR 1.05, 95% CI 1.01–1.10; *p* = 0.015) and MOF (OR 1.08, 95% CI 1.03–1.14; *p* = 0.001), but not with pneumonia or mortality (*p* > 0.05 respectively).

ISS was a strong univariable predictor for all adverse outcomes (*p* < 0.01 respectively).

Age was associated with an increased risk of pneumonia, MOF and mortality. A higher ASA class correlated with sepsis, pneumonia and mortality. Female sex was significantly associated with lower rates of sepsis (OR 0.48, 95% CI 0.26–0.90; *p* = 0.021).

### Multivariable analysis

Multivariable logistic regression models were fitted using complete-case analyses for the predefined adverse clinical outcomes ARDS, pneumonia, sepsis, MOF and mortality with a priori adjustment for BMI, age, sex, ASA and ISS. To reduce potential overfitting due to small event numbers, Firth’s penalized logistic regression was applied for the ARDS and MOF models. The results of these models are summarized in Table 3.

**Table 3:** Multivariable logistic regression for adverse clinical outcomes

Outcome	Predictor	Adjusted OR (95% CI)	p-Value
**ARDS**^**†**^	BMI	1.09 (1.01–1.16)	0.025*
	Age	1.00 (0.98–1.03)	0.718
	Female sex	0.31 (0.09–1.04)	0.057
	ASA	1.14 (0.69–1.88)	0.600
	ISS	1.06 (1.03–1.08)	< 0.001*
**Pneumonia**	BMI	0.99 (0.95–1.02)	0.435
	Age	1.02 (1.01–1.03)	< 0.001*
	Female sex	0.73 (0.51–1.04)	0.079
	ASA	1.25 (1.02–1.52)	0.031*
	ISS	1.06 (1.05–1.07)	< 0.001*
**Sepsis**	BMI	1.05 (1.00–1.10)	0.048*
	Age	1.01 (0.99–1.02)	0.463
	Female sex	0.49 (0.25–0.92)	0.028*
	ASA	1.34 (0.98–1.84)	0.070
	ISS	1.07 (1.05–1.09)	< 0.001*
**MOF**^**†**^	BMI	1.10 (1.04–1.16)	0.001*
	Age	1.04 (1.02–1.06)	< 0.001*
	Female sex	0.53 (0.23–1.11)	0.096
	ASA	0.92 (0.61–1.40)	0.712
	ISS	1.06 (1.04–1.08)	< 0.001*
**Mortality**	BMI	1.03 (0.99–1.08)	0.174
	Age	1.09 (1.08–1.11)	< 0.001*
	Female sex	0.78 (0.50–1.22)	0.274
	ASA	1.32 (1.00–1.75)	0.049*
	ISS	1.09 (1.07–1.11)	< 0.001*

^*^Statistically significant

^**†**^calculated using Firth’s penalized logistic regression analysis

BMI remained independently associated with ARDS (aOR 1.09 per kg/m^2^, 95% CI 1.01–1.16; *p* = 0.025), sepsis (aOR 1.05 per kg/m^2^, 95% CI 1.00–1.10; *p* = 0.048) and MOF (aOR 1.10 per kg/m^2^, 95% CI 1.04–1.16; *p* < 0.001) after adjustment for ISS, age, sex, and ASA. No significant independent association between BMI and pneumonia or mortality was observed.

Age was independently associated with MOF (aOR 1.04 per year, 95% CI 1.02–1.06; *p* < 0.001).

Female sex was independently associated with a lower risk of developing sepsis (aOR 0.48, 95% CI 0.25–0.92; *p* = 0.026).

Injury severity proved to be independently associated with all adverse clinical outcomes (*p* < 0.001, respectively).

In explorative sensitivity analyses including thoracic injury severity, with and without additional adjustment for ISS (ARDS ~ BMI + Age + Sex + ASA (+ ISS) + AIS Thorax), BMI remained independently associated with ARDS (aOR 1.079 (1.010–1.152), *p* = 0.024; aOR 1.082 (1.013–1.155), *s* = 0.018, respectively).

Model discrimination was acceptable/good for ARDS (AUC 0.78), pneumonia (AUC 0.76), sepsis (AUC 0.80), MOF (AUC 0.82) and excellent for mortality (AUC 0.90). Calibration assessed by the Hosmer–Lemeshow test showed adequate fit for all models (*p* > 0.05), except for pneumonia (*p* < 0.001), due to a high event rate. The Akaike information criterion indicated good model fit and parsimony for all models. No relevant multicollinearity was detected (all VIF < 2.0). Sensitivity analyses using Firth’s penalized logistic regression for ARDS and MOF produced effect estimates within ± 3% of those obtained with conventional logistic regression.

The categorical BMI models (Table 4) revealed that overweight patients did not experience significantly different risks than normal weight patients, however, they demonstrated a protective effect for sepsis (aOR 0.44).

**Table 4:** Comparison of categorical BMI effect with normal weight as reference

BMI category	Outcome	Adjusted OR (95% CI)	*p* value
Overweight (25–29.9 kg/m^2^)	ARDS	0.83 (0.34–1.99)	0.683
	Pneumonia	0.94 (0.68–1.31)	0.729
	Sepsis	0.44 (0.24–0.80)	0.008*
	MOF	0.98 (0.47–2.03)	0.962
	Mortality	1.02 (0.66–1.58)	0.923
Obesity I (30–34.9 kg/m^2^)	ARDS	2.82 (1.05–7.25)	0.032*
	Pneumonia	1.17 (0.72–1.87)	0.508
	Sepsis	2.07 (1.08–3.86)	0.024*
	MOF	2.93 (1.25–6.64)	0.011*
	Mortality	1.01 (0.52–1.88)	0.988
Obesity II–III (≥ 35 kg/m^2^)	ARDS	1.47 (0.22–5.83)	0.632
	Pneumonia	0.75 (0.34–1.50)	0.438
	Sepsis	2.29 (0.95–5.07)	0.051
	MOF	4.91 (1.74–12.75)	< 0.001*
	Mortality	1.74 (0.69–4.07)	0.221

Normal BMI (18.5–24.9 kg/m^2^) as reference category. Models adjusted for age, sex, ASA and ISS

^*^statistically significant

Obesity class I was associated with approximately threefold higher odds of ARDS and MOF and twice the sepsis risk. Patients with class II/III obesity (BMI ≥ 35 kg/m^2^) exhibited the highest risk of MOF (aOR 5.34).

## Discussion

In this large retrospective cohort of 1514 adult trauma patients, we found that a higher BMI was independently associated with an increased risk of ARDS, sepsis and MOF, but not pneumonia or mortality after adjustment for age, sex, ASA, and ISS. Categorical BMI analyses revealed a non-linear relationship, with overweight patients (BMI 25–29.9 kg/m^2^) showing neutral outcomes and a reduced risk of sepsis, whereas obesity (BMI ≥ 30 kg/m^2^) was associated with a marked increase in inflammatory and organ dysfunction–related complications. Injury severity remained the dominant predictor across all outcomes, yet BMI contributed independently to specific complications even after comprehensive adjustment. These findings demonstrate a distinct complication profile among obese trauma patients, suggesting heightened vulnerability to inflammatory and organ-failure sequelae while their mortality risk remains unaffected.

The associations we observed between BMI and adverse clinical outcomes are consistent with the concept of obesity amplifying the physiological response to severe trauma [34, 35]. The effects were consistent in all analyses for ARDS, sepsis and MOF, supporting the robustness of the observed associations.

In contrast, BMI was not independently associated with pneumonia, suggesting that localized infectious complications after trauma are more strongly driven by factors other than body composition.

Despite being associated with organ dysfunction, BMI was not independently associated with in-hospital mortality as a binary endpoint. This finding aligns with previous reports from trauma and critical care populations describing an “obesity paradox,” whereby higher BMI is associated with increased complication rates but not with higher mortality [14, 27]. Our cohort’s mortality was predominantly explained by injury severity, premorbidity (ASA) and age, underscoring the dominant role of the trauma burden and physiological reserve in determining survival.

Older age was also independently associated with pneumonia, MOF and mortality, somewhat reflecting the reduced physiological reserve and impaired immune response in elderly patients. These expected associations further support the internal consistency of the observed BMI associations.

Consistent with what we expected, injury severity emerged as the strongest and most consistent predictor across all adverse clinical outcomes. This finding supports the internal validity of our models.

All covariates included in the multivariable models were selected a priori based on clinical relevance. Their inclusion was intended to control for confounding rather than to establish independent causal relationships. While some covariates showed statistically significant associations with specific outcomes, the primary focus of this analysis was the independent effect of BMI on specific adverse clinical outcomes. Overall model discrimination was acceptable to good across all outcomes, with particularly strong discrimination for mortality. Calibration was adequate for most models, although statistical miscalibration was observed for the pneumonia model, likely reflecting the large sample size and higher event rate rather than poor clinical performance. Taken together, the model performance metrics support the robustness and interpretability of our findings.

Beyond the linear associations observed in the continuous BMI models, categorical analyses provide additional insight into clinically relevant threshold effects. In categorical analyses, overweight patients (BMI 25–29.9 kg/m^2^) did not show increased risks for adverse outcomes and even demonstrated a lower risk of sepsis, consistent with a limited obesity-paradox effect. In contrast, obesity was associated with substantially higher odds for ARDS, sepsis and multiorgan failure, indicating a clinically relevant threshold at a BMI of approximately 30 kg/m^2^. Severely obese patients exhibited the highest risk for multiorgan failure, suggesting a dose-dependent escalation of systemic vulnerability. Together, these findings support a non-linear relationship between BMI and post-traumatic complications. Interpretation of categorical BMI strata should, however, be undertaken with caution, as event numbers in higher obesity classes were limited. Continuous BMI models with consistent dose–response associations are statistically more robust. Therefore, categorical findings primarily illustrate potential threshold patterns rather than precise risk estimates for individual obesity classes.

BMI should be interpreted as a risk marker rather than a therapeutic target. It represents a crude surrogate and does not directly quantify body composition, muscle mass, or physiological reserve. Our study did not investigate BMI-targeted nutritional strategies. Accordingly, causal inferences regarding metabolic interventions cannot be drawn from this observational analysis.

### Comparison with literature

Our finding that BMI is independently associated with ARDS is consistent with prior studies identifying obesity as a risk factor for acute hypoxemic respiratory failure in critically ill or injured patients [8, 20, 36]. Obesity impairs lung compliance, reduces the functional residual capacity, and exacerbates ventilation–perfusion mismatch, thereby predisposing patients to respiratory decompensation [2]. Higher ARDS rates among obese individuals have been demonstrated in trauma populations [6, 36] with obesity being associated with nearly twice the risk of ARDS in severely injured patients, even after adjusting for confounders [5]. Our analysis corroborates these findings by demonstrating a continuous dose–response relationship between BMI and ARDS risk. Although thoracic injury severity is a known contributor to ARDS, sensitivity analyses including AIS thorax did not materially alter the BMI association, supporting robustness of the primary findings.

Similarly, the association between BMI and MOF aligns with data indicating that obesity amplifies systemic inflammatory responses through dysregulated adipokine signaling, chronic low-grade immune activation, and greater susceptibility to secondary insults [37]. Adipose tissue, especially visceral fat, acts as an active endocrine organ that produces pro-inflammatory adipokines (e.g., Leptin, TNF-α) while diminishing the secretion of protective anti-inflammatory adipokines (e.g., Adiponectin). This persistent low-grade inflammatory condition compromises the endothelium and systemic organs, hastening the progress to severe systemic inflammatory response syndrome (SIRS), sepsis and ensuing multi-organ failure after the traumatic "second hit" cascade [35].

Previous studies have linked obesity to higher SOFA scores, increased organ dysfunction and prolonged ICU stays following major trauma [5, 7, 19, 23, 27]. Our study confirms their evidence by showing that BMI is an independent predictor of sepsis and MOF. However, we failed to detect an independent association between BMI and mortality. This too is consistent with multiple large-scale analyses showing no significant increase in mortality among obese trauma patients and in some cases even lower adjusted mortality—often described as the “obesity paradox” [5, 7, 14]. Possible explanations include: (1) greater physiological reserve, where adipose tissue serves as an energy reservoir during the acute catabolic phase of trauma; (2) more aggressive early ICU management, or (3) methodological biases (e.g., collider stratification bias). Our findings suggest that although obesity predisposes patients to specific complications, these do not necessarily translate into higher mortality, particularly within modern multidisciplinary trauma care systems.

An unexpected finding in our cohort was the association between female sex and the lower odds of contracting sepsis. This evidence, however, is consistent with previously reported sex-related differences in immune and inflammatory responses following trauma, and suggest that sex may act as a biological modifier of post-traumatic inflammation rather than a major determinant of overall outcome [38, 39].

Interestingly, BMI did not prove to be independently associated with pneumonia after adjustment. Prior studies have reported mixed results, with some suggesting a higher infection risk in obese trauma patients [7, 40, 41], while others found no independent effect after accounting for comorbidities and injury severity [8, 28]. In our fully adjusted models, this likely reflects the dominant influence of age and injury severity on infectious complications. Moreover, pneumonia is strongly influenced by post-injury course–related factors such as intubation, tracheostomy, and the duration of mechanical ventilation, which were not included in our a priori patient- and injury-based multivariable models.

### Clinical implications

Our findings have implications primarily for risk stratification and clinical vigilance in obese trauma patients. Clinicians should be aware of the higher observed risk of ARDS, sepsis, and multiorgan failure in obese trauma patients and may consider heightened monitoring for early signs of organ dysfunction. However, our data do not support fundamental alterations of established trauma care protocols. Despite the higher vulnerability of obese patients towards the aforementioned serious adverse clinical outcomes, current ICU treatment strategies seem to be successful in keeping mortality rates at a BMI-independent rate.

### Strengths and limitations

This study benefits from a very large single-center sample size of a contemporary trauma population and multiple analytical approaches including multivariable adjustment for key confounders as its main strengths.

Several limitations should be acknowledged. First, the retrospective design precludes causal inference and is subject to residual confounding despite multivariable adjustment. Second, BMI served as a surrogate measure of body composition and does not account for fat distribution, which may be more relevant determinants of outcome [42]. Third, although the events-per-variable ratio was low for ARDS and borderline for MOF, adaptation of the logistic regression model to Firth’s penalized analysis should make fundamental overfitting-driven bias unlikely. Nevertheless, some degree of effect size inflation cannot be fully excluded.

Although ASA classification served as a global marker of baseline health status, it may not fully capture the heterogeneity and differential impact of individual comorbid conditions. Therefore, residual confounding by obesity-associated comorbidities cannot be excluded.

Frailty and pre-injury functional status were not systematically documented and could not be included in the analysis. Especially in elderly patients, BMI may not adequately reflect physiological reserve, as sarcopenic obesity may be present. Residual confounding by frailty therefore cannot be excluded.

Mortality was assessed as a binary in-hospital outcome without time-to-event analysis. Data on withdrawal or limitation of life-sustaining therapy were not available, which may influence survival estimates independently of BMI.

Treatment-related factors such as transfusion burden, fluid resuscitation, and ventilation parameters were not included in the primary models. As these variables may lie on the causal pathway between injury severity, obesity-related physiological response and ARDS development, adjustment could introduce overadjustment bias. Therefore, they were not considered confounders in the main analysis.

As a single-center study conducted at a high-volume Level I trauma center, generalizability of the results is limited. Although this design ensures consistent documentation and uniform application of outcome definitions, case-mix and management strategies may differ across institutions. Therefore, our findings require validation in independent multi-center cohorts.

## Conclusions

A higher BMI was independently associated with ARDS, sepsis and MOF, but no independent association with mortality was detected in this cohort of adult trauma patients. This suggests that BMI may function as a clinically relevant risk marker.

## Supplementary Information

Supplementary Material 1.

## Data Availability

The anonymized dataset that supports the findings of this study is available from the corresponding author upon reasonable request.

## Abbreviations

aOR: Adjusted Odds Ratio
ARDS: Acute Respiratory Distress Syndrome
ASA: American Society of Anesthesiologists
AIC: Akaike Information Criterion
AUC: Area Under the receiver operating characteristic Curve
BMI: Body Mass Index
CI: Confidence Interval
CT: Computed Tomography
HL: Hosmer–Lemeshow goodness-of-fit test
ICU: Intensive Care Unit
ISS: Injury Severity Score
MOF: Multi-Organ Failure
OR: Odds Ratio
SD: Standard Deviation
STROBE: Strengthening the Reporting of Observational Studies in Epidemiology
VIF: Variance Inflation Factor
WHO: World Health Organization
RECORD: REporting of studies Conducted using Observational Routinely-collected Data
