# Supplementary material for: BMI is independently associated with ARDS, sepsis and multiorgan failure after major trauma—results of a high-volume retrospective observational cohort study
Source: Scand J Trauma Resusc Emerg Med. 2026 Mar 27;34:69. doi: 10.1186/s13049-026-01603-7 (PMC13063989; doi:10.1186/s13049-026-01603-7)
Supplement: Supplementary file 1 — Supplementary Material 1. [file 13049_2026_1603_MOESM1_ESM.docx]

**Supplementary Table S1: Univariable Logistic Regression Analyses of Patient Characteristics as predictors for Adverse Clinical Outcomes.**

| **Outcome** | **Predictor** | **OR (95% CI)** | **p value** |
| --- | --- | --- | --- |
| **ARDS** | BMI (per kg/m²) | 1.08 (1.02–1.15) | 0.012* |
|  | Age (per year) | 1.01 (0.99–1.03) | 0.383 |
|  | Female sex | 0.31 (0.09–1.02) | 0.054 |
|  | ASA (per class) | 1.26 (0.83–1.90) | 0.280 |
|  | ISS (per point) | 1.05 (1.03–1.08) | <0.001* |
|  | AIS Head/Neck/Cervical spine (per point) | 1.26 (1.03–1.54) | 0.025* |
|  | AIS Face (per point) | 0.83 (0.54–1.28) | 0.403 |
|  | AIS Thorax/Thoracic spine (per point) | 1.60 (1.24–2.07) | <0.001* |
|  | AIS Abdomen/Lumbar spine (per point) | 1.24 (0.98–1.57) | 0.075 |
|  | AIS Pelvis/Extremities (per point) | 1.16 (0.88–1.52) | 0.287 |
|  | AIS External (per point) | 1.58 (0.99–2.51) | 0.053 |
| **Pneumonia** | BMI (per kg/m²) | 1.00 (0.97–1.03) | 0.934 |
|  | Age (per year) | 1.03 (1.02–1.03) | <0.001* |
|  | Female sex | 0.82 (0.59–1.14) | 0.244 |
|  | ASA (per class) | 1.49 (1.27–1.76) | <0.001* |
|  | ISS (per point) | 1.06 (1.05–1.07) | <0.001* |
|  | AIS Head/Neck/Cervical spine (per point) | 1.29 (1.20–1.39) | <0.001* |
|  | AIS Face (per point) | 1.21 (1.07–1.37) | 0.002* |
|  | AIS Thorax/Thoracic spine (per point) | 1.62 (1.47–1.79) | <0.001* |
|  | AIS Abdomen/Lumbar spine (per point) | 1.30 (1.18–1.43) | <0.001* |
|  | AIS Pelvis/Extremities (per point) | 1.18 (1.08–1.30) | <0.001* |
|  | AIS External (per point) | 1.12 (0.97–1.29) | 0.120 |
| **Sepsis** | BMI (per kg/m²) | 1.05 (1.01–1.10) | 0.015* |
|  | Age (per year) | 1.01 (1.00–1.02) | 0.052 |
|  | Female sex | 0.48 (0.26–0.90) | 0.022* |
|  | ASA (per class) | 1.38 (1.06–1.78) | 0.016* |
|  | ISS (per point) | 1.07 (1.05–1.08) | <0.001* |
|  | AIS Head/Neck/Cervical spine (per point) | 1.41 (1.22–1.64) | <0.001* |
|  | AIS Face (per point) | 1.11 (0.89–1.38) | 0.355 |
|  | AIS Thorax/Thoracic spine (per point) | 1.59 (1.34–1.89) | <0.001* |
|  | AIS Abdomen/Lumbar spine (per point) | 1.52 (1.28–1.81) | <0.001* |
|  | AIS Pelvis/Extremities (per point) | 1.32 (1.12–1.56) | 0.001* |
|  | AIS External (per point) | 1.21 (0.86–1.70) | 0.272 |
| **MOF** | BMI (per kg/m²) | 1.08 (1.03–1.14) | 0.001* |
|  | Age (per year) | 1.03 (1.02–1.05) | <0.001* |
|  | Female sex | 0.64 (0.31–1.33) | 0.234 |
|  | ASA (per class) | 1.42 (1.02–1.98) | 0.037* |
|  | ISS (per point) | 1.06 (1.04–1.08) | <0.001* |
|  | AIS Head/Neck/Cervical spine (per point) | 1.49 (1.22–1.82) | <0.001* |
|  | AIS Face (per point) | 1.21 (0.93–1.58) | 0.157 |
|  | AIS Thorax/Thoracic spine (per point) | 1.46 (1.20–1.78) | <0.001* |
|  | AIS Abdomen/Lumbar spine (per point) | 1.48 (1.24–1.77) | <0.001* |
|  | AIS Pelvis/Extremities (per point) | 1.35 (1.08–1.67) | 0.007* |
|  | AIS External (per point) | 1.31 (0.86–1.98) | 0.203 |
| **Mortality** | BMI (per kg/m²) | 1.02 (0.99–1.06) | 0.227 |
|  | Age (per year) | 1.08 (1.07–1.10) | <0.001* |
|  | Female sex | 1.17 (0.81–1.69) | 0.398 |
|  | ASA (per class) | 2.51 (2.02–3.11) | <0.001* |
|  | ISS (per point) | 1.06 (1.05–1.08) | <0.001* |
|  | AIS Head/Neck/Cervical spine (per point) | 1.93 (1.72–2.16) | <0.001 |
|  | AIS Face (per point) | 1.15 (0.98–1.36) | 0.087 |
|  | AIS Thorax/Thoracic spine (per point) | 1.02 (0.92–1.13) | 0.752 |
|  | AIS Abdomen/Lumbar spine (per point) | 0.97 (0.85–1.11) | 0.659 |
|  | AIS Pelvis/Extremities (per point) | 0.80 (0.70–0.92) | 0.001* |
|  | AIS External (per point) | 1.05 (0.80–1.39) | 0.708 |

All analyses are univariable binary logistic regression models without adjustments for confounders. AIS = Abbreviated Injury Scale. *Statistically significant
